# The Complex Structure of Receptive Fields in the Middle Temporal Area

**DOI:** 10.3389/fnsys.2013.00002

**Published:** 2013-03-06

**Authors:** Micah Richert, Thomas D. Albright, Bart Krekelberg

**Affiliations:** ^1^The Salk Institute for Biological StudiesLa Jolla, CA, USA; ^2^Center for Molecular and Behavioral Neuroscience, Rutgers UniversityNewark, NJ, USA

**Keywords:** receptive field, motion, middle temporal area, direction tuning, visual perception

## Abstract

Neurons in the middle temporal area (MT) are often viewed as motion detectors that prefer a single direction of motion in a single region of space. This assumption plays an important role in our understanding of visual processing, and models of motion processing in particular. We used extracellular recordings in area MT of awake, behaving monkeys (*M. mulatta*) to test this assumption with a novel reverse correlation approach. Nearly half of the MT neurons in our sample deviated significantly from the classical view. First, in many cells, direction preference changed with the location of the stimulus within the receptive field. Second, the spatial response profile often had multiple peaks with apparent gaps in between. This shows that visual motion analysis in MT has access to motion detectors that are more complex than commonly thought. This complexity could be a mere byproduct of imperfect development, but can also be understood as the natural consequence of the non-linear, recurrent interactions among laterally connected MT neurons. An important direction for future research is to investigate whether these in homogeneities are advantageous, how they can be incorporated into models of motion detection, and whether they can provide quantitative insight into the underlying effective connectivity.

## Introduction

From the time that Hartline (1938) first introduced the concept of a receptive field, it has strongly influenced thinking about processing in the visual system. For instance, the idea that an early visual neuron responds only to a small, contiguous subset of visual space directly leads to a modular view of early visual processing in which neurons neatly tessellate visual space. The receptive field concept can be extended quite naturally from the spatial domain to arbitrary feature dimensions; retinal ganglion cells have spatial receptive fields, V1 neurons additionally have an orientation receptive field, and neurons in the middle temporal area (MT) have a spatial as well as a directional receptive field – the range of motion directions to which the neuron responds.

In area MT, a common view is that neurons prefer the same direction throughout their spatial receptive field, and that sensitivity is high at one location, but drops off toward the edges of the receptive field. We will refer to this as a homogeneous receptive field. At a relatively coarse scale, experimental data (Raiguel et al., 1995) support this view, and this has led to models of motion processing that take this view quite literally. Most models, for instance, assume receptive fields that are Gaussian in space and with a single direction preference (Royden, 1997; Simoncelli and Heeger, 1998; Rust et al., 2006). The view of a homogenous receptive field is theoretically appealing because it identifies MT neurons as elementary motion detectors whose sole task it is to detect a single direction of motion in a single part of visual space. Other cortical areas such as the Medial Superior Temporal area (MST) can then perform more complex motion analyses by judiciously combining the elementary motion detectors of area MT.

Given the near universal adoption of this view, a rigorous test is important. We developed a reverse correlation method to quantitatively map the receptive fields in the spatial and direction domain and used it to map the fine structure of the receptive field of neurons in area MT. To our surprise, we found that the assumptions of spatial and directional homogeneity were often violated in MT neurons. A quarter of MT neurons had spatial receptive field profiles with multiple peaks separated by regions of low sensitivity. Even more strikingly, in nearly 40% of MT neurons direction preference varied with spatial position. These findings have significant implications for the complexity of motion analysis that individual MT neurons can perform.

## Materials and Methods

We recorded from two adult male rhesus monkeys (*Macaca mulatta*), weighing 8.5–9.5 kg. Experimental protocols were approved by the local animal use committees, and conform to the National Institutes of Health guidelines for humane care and use of laboratory animals.

### Surgical preparation

A head post and a recording cylinder were affixed to the skull using stainless steel rails, screws, and dental acrylic. We estimated the anatomical location of area MT from structural MR scans and centered the recording chambers vertically above this area. The coordinates of the chambers were 4 mm posterior and 19 mm lateral in the first animal, and 4 mm posterior and 20 mm lateral in the second animal. All surgical procedures were performed under sterile conditions using isoflurane anesthesia.

### Visual stimuli

All visual stimuli were generated with an OpenGL-based library (Neurostim: http://sourceforge.net/projects/neurostim) using an ATI Radeon 9600 graphics card connected to a 21″ analog RGB video monitor (either Sony GDM-2000TC or Sony GDM-C520). We used refresh rates of 75, 120, and 150 Hz. The screen resolution was always 1024 by 768 pixels. Monkeys viewed the display binocularly from a distance of 57 cm in a dark room (<0.5 cd/m^2^); stimuli had zero binocular disparity. The monitor subtended 40° by 30°.

At the start of each trial, a red fixation dot (0.1° diameter) appeared. The fixation dot was normally located at the center of the screen, but could be moved to more eccentric locations so that the visual stimulus would stimulate more of a peripheral receptive field. Once the monkey had maintained its gaze on the fixation target for 100 ms, the visual stimulus appeared and was shown for up to 3 s.

We used three kinds of visual stimuli. The first of these was a novel stimulus specifically designed to map receptive fields using reverse correlation and obtain high resolution in both the spatial and direction domain. The other two stimuli were used in conjunction with more traditional analysis methods and served to validate elements of the reverse correlation mapping approach. Detailed properties of these stimuli are given here:

#### Brownian motion dots

##### Goal: to map the fine structure of the receptive field

The Brownian Motion dots (BMDots) stimulus was composed of 300 independently moving dots with unlimited lifetime (0.25 dots per °^2^). Each dot was rendered with a Gaussian 2D luminance profile, with a sigma of 0.2°. The Gaussian was clipped at 2.5 standard deviations. The dots moved at a constant 8°/s. Dots moved in their assigned direction for 200 ms, after which a new direction was chosen from a uniform circular distribution. The direction changes were interleaved such that only a portion of the dots changed direction on any one frame. The background was mean gray (18.3 cd/m^2^), and the contrast between the center of the white dots and the background was 0.25. When two or more dots overlapped, their RGB values were summed; given the relatively low contrast of each dot, this approximates linear summation of dot luminance.

#### Grid receptive field

##### Goal: to confirm the spatial profile of the receptive field

The stimulus was structured on a grid subtending 11° × 11° and centered on the classical RF. Individual grid patches were 1° × 1°. A single dot moved in a constant direction inside one of the 121 grid patches for 200 ms, disappeared, and immediately reappeared at a new location. When the dot reached the edge of the grid patch, it wrapped around to the other side of the patch. All other aspects of the stimulus (background, dot size, speed, luminance, etc) were identical to those of BMDots. Due to the small size of the single moving dot, the responses evoked by this stimulus were relatively small for MT units (40 Hz average peak firing rate), but they were reliably direction selective.

#### Preferred direction

##### Goal: to map the direction preference in a subset of spatial locations inside the RF

This stimulus was identical to the Grid Receptive Field (GridRF) stimulus, except that we pre-selected up to 5 of the 121 patches of the grid for this high resolution direction mapping. A location was chosen at random from these five grid patches and the single dot moved within that patch in one of 64 directions for 200 ms, then a new direction and location were randomly chosen. All other stimulus attributes matched the Grid RF and BMDots stimulus.

### Electrophysiological procedures

We used tungsten [lacquer coated (FHC, Bowdoin, ME, USA) and glass coated (Alpha Omega, Nazareth, Israel)] microelectrodes with 1–4 MΩ impedance (measured at 1 kHz) and used the Plexon System (Plexon Inc., Dallas, TX, USA) to filter, store, and sort the signals. The electrodes were positioned using a hydraulic micropositioner (David Kopf, Tujunga, CA, USA; model 650).

We identified area MT by means of physiological and anatomical criteria: strong directionally selective responses, RFs that were relatively small compared to those of neighboring area MSTd, and recording locations on the posterior bank of the superior temporal sulcus (i.e., after traversing a sulcus in the dorsal to ventral electrode approach). The chamber coordinates and depths of the recorded neurons were consistent with the expected location of area MT derived from structural MRI scans.

Spikes were detected using a box sorter at the time of the experiment, but re-sorted offline using the principal components of their waveforms (Offline Sorter, Plexon, Inc). Strict criteria were used to define single units: for a unit to be considered isolated the projection of its waveform onto the first and second principal components had to be (visually) well separated from the projections of all other waveforms recorded on that electrode. Moreover, we required that few spikes (=0.1%) had an inter spike interval less than 1 ms. For all example cells shown in figures there were 0 spikes less than 0.9 ms apart. Most recordings of single cells lasted at least 30 min. Over the recording time the waveforms normally changed, however only the time range for which they were separated from the noise and other units were used in the analysis.

We validated these qualitative selection criteria *post hoc* by determining the *L*-ratio. The *L*-ratio is a measure of the inverse of the distance between spikes belonging to one cluster (i.e., a putative neuron) and all spikes belonging to other clusters (including the noise; Schmitzer-Torbert et al., 2005). These distances were calculated in the feature space defined by the first two principal components of the waveforms. Colloquially, a low *L*-ratio indicates that there is a good “moat” around the putative neuron’s spikes in feature space, and it is therefore well-isolated from the noise and other spikes on the same electrode (Schmitzer-Torbert et al., 2005).

### Recording procedure

The animals sat in a standard primate chair (Crist Instruments, Germantown, MD, USA) and their head movements were constrained by a head post. The behavioral paradigm was controlled using the NIMH CORTEX program (VCortex 1.1 running under Windows 98 or VCortex 2.1 running under Windows 2000).

We monitored eye position using an infrared video-based device (ISCAN, Burlington, MA, USA) with temporal resolution of 120 Hz, and a nominal spatial resolution of 30 min of arc. The animals fixated a red dot for the duration of each trial. Fixation within a 1° radius around the fixation target was rewarded with a small drop of juice at the end of each trial. Failure to maintain fixation terminated the trial and we discarded data obtained while the animal’s eye position was outside the fixation window. In a typical session, the standard deviation of the actual eye position was <0.15°. An analysis in which the measured eye position at each time point was taken into account to calculate the retinal stimulus motion led to more blurred spike-triggered averages (STA; not shown) than the analysis we present here, which assumes eye position to be constant.

While slowly advancing the electrode into the brain, we presented a full-field stimulus that moved in all directions over time (Schoppmann and Hoffmann, 1976; Krekelberg, 2008). The background hash of area MT responds to this stimulus with an easily identifiable direction selective response. We advanced the electrode until one or more direction selective single units could be isolated. Once isolation had been achieved, we presented the BMDots stimulus and started the online analysis. As soon as sufficient data had been collected for a stable estimate of the RF using reverse correlation (∼10 min), the control stimuli were added to the stimulus presentation paradigm. Typically, the Grid RF stimulus was added first, followed by the Preferred Direction (PrefDir) stimulus. For the remainder of most sessions all stimuli were then randomly interleaved. In a subset of sessions we monitored convergence of the tuning estimates and once those were stable for more than 20 trials, we removed that stimulus from the set to provide more presentation time for the remaining stimuli.

### Data analysis

#### Reverse correlation

**Figure 1:**
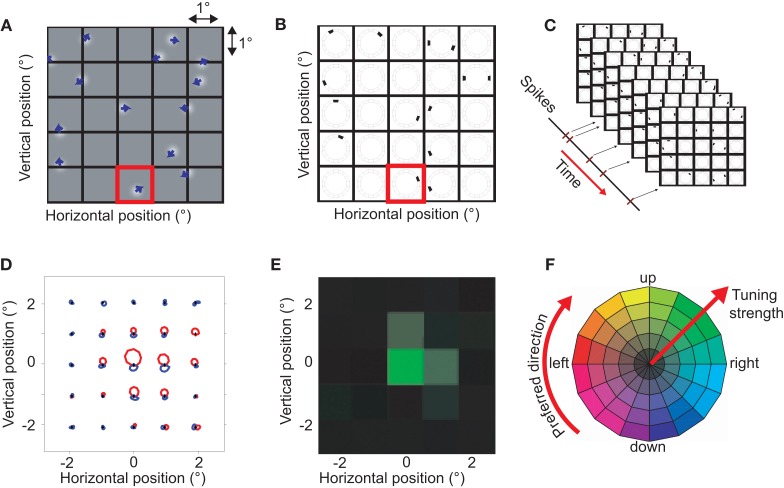
**The BMDots stimulus, its analysis, and representation of the results**. The BMdots stimulus consisted of 300 independently moving dots filling the entire monitor. **(A)** The spatial grid. To construct a discrete stimulus representation, we imposed a spatial grid, here shown with a resolution of 1 × 1 degree of visual angle. **(B)** The space-direction histogram. For each frame, the directions of motion within a grid were binned. For instance, the single dot that moved up and to the right in the (red) highlighted grid location in **(A)**, resulted in the direction histogram shown in the corresponding location in **(B)**. Note that, for illustration purposes, only 12 directional bins are shown here. The collection of direction histograms for all spatial locations is called the space-direction histogram. **(C)** We determined the spike-triggered average (STA) of the stimulus by performing a cross-correlation with a fixed lag (see Materials and Methods) between the neural response and the sequence of space-direction histograms. **(D)** The STA space-direction histograms for 25 spatial grid locations for one example neuron. This representation shows the complete direction tuning curve at each grid location. Red represents excitatory directions, blue suppressive (see Materials and Methods; **E)** For simplicity of the visual representation, we reduced the STA histograms of **(D)** to a single preferred direction (represented as a hue), and the strength of the direction preference (represented as color saturation). This example neuron had a strong preference for upward motion in one spatial location (green) and a weak preference for that same direction of motion in two neighboring locations (pale green). **(F)** The color wheel used to represent preferred direction and strength of the direction tuning. High saturation corresponds to highly direction selective locations.

The goal of the BMDots stimulus was to map direction preference with high resolution in the spatial and direction domain. In brief, the analysis consisted of three steps. First, we binned time and subtracted onset and adaptation response components unrelated to local direction preferences to calculate what we refer to as the selective response. Second, we binned the continuous BMDots stimulus in space (Figure 1A) and direction (Figure 1B). Third we performed reverse correlation between the binned stimulus representation and the selective response (Figure 1C).

##### The binned selective response

We binned the spike trains using bins equal to the duration of a single monitor refresh frame and then estimated the trial-locked, non-selective response of the cell by averaging all trials together per time bin. We smoothed this average temporal signal using a Gaussian filter (10 ms width) and subtracted this signal from the response in every trial. This procedure reduced the contaminating influence of onset transients and other adaptation effects that occurred on the time scale of a single trial (Priebe et al., 2002; Krekelberg et al., 2006a; Schlack et al., 2007). Additionally, we subtracted the mean firing rate in each trial from the response in that trial. This reduced the influence of adaptation that took place on a time scale longer than a single trial. These preprocessing steps substantially improved our signal to noise ratio and led to cleaner RF estimates, but there were no qualitative differences in the maps obtained with or without this preprocessing (not shown).

##### The stimulus space-direction histogram

The BMDots stimulus was continuous in space and direction, but for the reverse correlation we imposed an arbitrary grid of 1° × 1° patches on the 39° × 29° monitor screen (Figure 1A). For each frame in the stimulus movie and each patch in the grid, we calculated the instantaneous direction of motion for each of the dots inside the patch. These directions were binned in 64 direction bins and the histogram of directions (number of dots per direction) was used as a representation of the stimulus direction in a patch for the given frame (Figure 1B). This binning procedure allowed us to represent the stimulus in a given frame as a three-dimensional matrix (referred to as a space-direction histogram); two dimensions correspond to the spatial position, and the third to the direction of motion. The numbers in this matrix represent the number of dots within a 1° × 1° spatial patch moving in a particular direction (see Figure 1B).

##### The spike-triggered average

To calculate the STA, we calculated the cross-correlation between the binned selective response and the space-direction histogram for a range of assumed latencies (0–100 ms). Given the assumptions underlying reverse correlation analysis, such an STA can be interpreted as a complete direction tuning curve (in 64 bins) for each 1 × 1° patch on the screen. In Figure 1D we show the tuning curves per patch. We compared the STA per direction and per patch to a baseline STA that consists of the mean across all patches. This allowed us to separate excitatory directions and suppressive directions. Excitatory directions are those stimulus directions that are followed by more than the average number of spikes; they are represented in red in Figure 1D. Suppressive directions are stimulus directions that are followed by less than the average number of spikes (blue in Figure 1D). In addition, this baseline subtraction allowed us to detect patches in which the response changed in a direction independent manner.

To simplify the analysis, we reduced the tuning curves to single vectors per patch by determining the vector sum. This is a measure of a neuron’s preferred direction and its tuning strength per patch and will be referred to as the Preferred Direction vector (PD). Because the vector sum removes any contributions that are not tuned for direction, this measure of tuning strength is independent of the preprocessing steps that removed such contributions.

Separately for each cell, we selected the optimal latency for the spike-triggered averaging process by determining which latency led to the largest preferred direction vectors summed over all patches. The PD corresponding to this latency was used for all subsequent analyses for this cell. Please note that due to the slow changes in stimulus direction (every 200 ms), the temporal profile of the STA was smooth and extended far in time. As a consequence, variation in the choice of this latency on the order of tens of milliseconds did not affect our analyses qualitatively. This stimulus limitation also prevented us from investigating possible differences in latency across the RF.

##### Validation

The reliability of reverse correlation analyses depends on the sample statistics of the stimulus distribution as well as the number of spikes that enter the analysis. The number of spikes in our analyses ranged from 472 to 120,364 with a median of 8156. For each of the example cells in the Figures, the number of spikes is listed. To validate our results, we determined the STA on a random subset of 50% of the data, and compared it with the STA of the remaining 50%. To quantify similarity, we determined an index given by the inner product of the two normalized STA vectors. This index can range from −1 to 1, where 1 indicates perfect agreement. In our dataset the similarity ranged from 0.66 to 1.00 with a median similarity of 0.90. The high degree of reproducibility based on independent samples shows that the STA reliably identifies the spatial and direction tuning properties of the neurons.

#### Statistical analysis

##### Null distribution

To create a null distribution of the STAs we calculated the STA after reversing the response of the cell in time and then performing the standard STA calculation procedure, described above. The reversal in time was chosen to destroy stimulus response correlations (i.e., by using non-causal correlation delays), while keeping the statistics of the stimulus (the distribution of motion vectors) and spike counts intact. For a subset of cells we also calculated null distributions based on randomly generated stimulus sequences; this did not affect the results. We pooled the resulting values over all stimulus patches to create a null distribution; i.e., the distribution of values of the STA that would be expected if there were no correlation between the spikes and the stimulus. To determine significance of directional tuning strength, we calculated a distribution of null PDs from these null STAs. This distribution of (squared) vector lengths follows a χ^2^ distribution whose variance we estimated from the distribution of null PDs.

##### Receptive field

Per patch, we calculated the vector length of the PD and compared it to the χ^2^ distribution determined from the null STA (see above). Patches with significant (*p* < 0.01) direction tuning were considered part of the RF. This significance test was applied to each of the ∼1100 patches. To correct for the resulting increase in type I errors due to multiple comparisons, we included only those patches that had three or more statistically significant neighbors (Forman et al., 1995). We found no patches that modulated the firing rate of a neuron in a direction independent manner. In other words, because every patch that was visually responsive was also directionally tuned, the RF effectively consisted of all patches with a visual response.

##### Selecting excitatory regions

A particular direction (in a particular patch) was considered excitatory if its value in the STA was higher than the mean value in the null STA. Similarly, a suppressive direction was identified as a direction that had a lower value in the STA than the mean value in the null STA. We considered a whole patch to be suppressive if there was greater suppression than excitation when summed across all directions. Because we were interested in the excitatory receptive field, and not the suppressive surround, these suppressive patches were excluded from analysis. Typically this excluded less than a quarter of the patches from analysis (median percentage of suppressive patches 22.5%).

##### Multiple directions

The standard test to compare two circular variables (the Watson–Williams test) is not applicable to two circular variables with small and potentially different lengths of the vector sum. Moreover, our data are multivariate (composed of a direction and response magnitude) while Watson–Williams and other standard circular tests are univariate. We therefore used a bootstrap test (below), to perform pair-wise comparisons between all significantly tuned patches within the receptive field. A cell in which one or more patches were found to have significantly different preferred directions was considered to have multiple preferred directions.

##### Multiple peaks

In a single-peaked RF the response along any line drawn from the most responsive patch to any other part of the RF should decrease monotonically. To test the null hypothesis of a monotonic RF, we defined the RF conservatively by using a strict criterion for patches to be included in the RF (direction tuning test passed at *p* < 0.005 instead of *p* < 0.01). As before, we excluded locations dominated by suppression to exclude the spatially disjoint suppressive surround. We then drew lines from the peak to all other patches in the receptive field; the number of such lines varied between 1 and 85 with a median of 12 per neuron. For each pair of patches that such a line intersected, we used the bootstrap test (below) to determine whether the response in the farther patch was smaller than that of the closer patch. The number of patches thus compared varied between 1 and 1307, with a median of 18 per neuron. The bootstrap percentile value was Bonferroni-corrected for these multiple comparisons. In addition to the test for monotonicity, we also counted the number of peaks in the RFs directly by thresholding the STA, and counting the number of isolated regions.

##### Bootstrap

We used a percentile bootstrapping technique (Wilcox, 2004) to compare direction tuning (*multiple preferred directions*) and/or tuning strength (*multiple peaks*) between patches within the receptive field.

For each patch we had a set of polar data: motion directions and corresponding responses (the raw data used to calculate the STA at one patch). To determine differences in preferred direction (while ignoring differences in tuning strength), we normalized the polar data per patch to sum to a vector length of one. To determine differences in tuning strength (while ignoring differences in direction preference), we rotated the two datasets such that their STAs had the same preferred direction.

We then created a null distribution by combining the data points from patch one (N1 data points: the motion vectors of the dots in 200 ms of data as this is the timescale at which the stimuli are independent) and from patch two (N2: the motion vectors of the dots moving within patch two in the 200 ms period). From this combined distribution we randomly sampled (with replacement) a set of N1 and a set of N2 data points to simulate data under the null hypothesis that the distributions were identical. For the direction test, we then determined the angular difference between the preferred directions of the two simulated data sets. For the tuning strength test, we determined the difference between the summed vector lengths of the two simulated data sets. This was repeated many times to estimate the cumulative distribution that would be expected if the data from patch 1 and patch 2 came from the same underlying distribution. The number of repeats was adjusted (up to 10 million) to obtain a reliable estimate of the Bonferroni-corrected cut-off percentile. The value for either the angular difference or the vector length difference between patch 1 and patch 2 was then compared to the Bonferroni-corrected 95th percentile of the cumulative null distribution.

#### Control stimuli

The online analysis of responses to the BMDots stimulus allowed us to select spatial locations and directions to probe with the control stimuli. Unless otherwise noted, we analyzed the response to control stimuli (GridRF and PrefDir) by simply averaging the firing rate in a 200 ms response window after stimulus onset, adjusted for the onset latency. This onset latency was estimated as the first time at which the neuron’s response was five standard deviations above or below baseline. The same (shortest) latency value across patches was used for all patches. Note that the firing rates were not preprocessed to remove non-tuned responses and adaptation effects (see Reverse Correlation analysis, above) and therefore provide a direct estimate of actual average firing rates.

For the PrefDir stimulus, we used the online analysis of the BMDots stimulus to select the most responsive patch and up to four other patches. These other patches were only included if their preferred direction differed significantly (*p* < 0.01) from the preferred direction of the most responsive patch. We calculated the mean firing rate per location and direction, adjusted for response latency. These mean responses were smoothed using a Gaussian kernel with a standard deviation of 5° to estimate a tuning curve per patch.

#### Relative tuning strength and size

For each cell with at least two preferred directions, we wished to compare the visual drive from each of these regions. To do this, we first separated the RF into two sub-regions, and then calculated the tuning strength in each of these regions. Specifically, we first constructed – for each cell – a preferred direction similarity matrix for all patches in the RF. Each entry in this similarity matrix (*S_ij_*) was the *p*-value of the bootstrap test that compared the preferred direction in patch *i* with patch *j*; this reflects the extent to which the preferred direction in each of the patches was statistically similar. Based on this matrix, we divided the receptive field into two regions (using *K*-means clustering with *K* = 2). Note that this analysis would not give meaningful results for neurons with a single preferred direction across the RF; hence the analysis shown in Figure 4 was restricted to neurons that passed the statistical test for multiple preferred directions. Given this pre-selection it is not surprising that the sub-regions had different preferred directions (i.e., the distribution in Figure 4A should not be centered on zero); the histogram only serves to quantify the size of the effect. We then determined the summed vector length (*R*) by summing the PD vectors (see above) across the patches of each sub-region. The summed vector length represents a measure of the tuning strength of the neuron from each region. The region with the largest sum is referred to as the primary region; the other region is the secondary region. We defined the relative tuning strength as *R*_secondary_/*R*_primary_ × 100%. This quantifies the drive from the secondary region as a percentage of the primary region; we refer to it as the relative tuning strength. The analogous calculation based on the number of patches (*N*) in the primary and secondary region resulted in the relative size measure: *N*_secondary_/*N*_primary_ × 100%.

#### Valley-over-peak analysis

Based on our definition of the RF (See Receptive Field, above), we identified the location with the strongest response (primary peak) and the convex hull of the RF. We then set a response threshold and lowered it from the primary peak value until a second, isolated peak appeared within the convex hull of the RF (secondary peak). A secondary peak could be identified with this method for 93 cells. The valley was defined as the location on the line between the primary and secondary peaks at which the tuning strength was weakest. The valley-over-peak ratio was defined as the ratio between the tuning strength at the valley and the tuning strength at the secondary peak (See Figure 10A).

#### Similarity index

We defined a similarity index as the inner product of two normalized vectors. For the direction mapping, this was the inner product of the direction tuning curve as mapped by PrefDir and the direction tuning curve mapped with BMDots. For the spatial mapping, we compared the response vector from the GridRF paradigm with the tuning strength determined with the BMDots stimulus. This similarity index is bounded by −1 and 1, with 1 representing a perfect match.

## Results

We recorded from 151 well-isolated single cells from two monkeys (Monkey S: 73, Monkey M: 78). The retinal eccentricity of the center of these neurons’ receptive fields ranged from 0.9 to 18.3° (mean: 7.4°).

Based on responses to a stimulus consisting of 300 dots that moved independently in a Brownian motion fashion across the whole screen, we estimated both the spatial extent of the RF and the preferred direction at each spatial location using reverse correlation (BMDots; see Materials and Methods). We analyzed only those spatial locations where the response was significantly direction-tuned and dominated by excitation (see Materials and Methods). To validate this spike-triggered analysis we confirmed that receptive field estimates based on the first and second half of the data were highly similar; the median similarity index (See Materials and Methods) was always above 0.66 and the median across the sample of 151 neurons was 0.9.

About half of the MT cells preferred the same direction of motion throughout a contiguous region of space. These receptive fields conform to the expectation in area MT; we refer to them as “simple” receptive fields. The other half of MT cells, however, deviated significantly from the conventional view of an MT receptive field; the preferred direction changed across the RF, and there were clear gaps in the RF where direction-tuned visual responses were much harder to evoke than in neighboring locations. We refer to these unexpected receptive fields as “complex.” Please note that the simple/complex nomenclature we use here for MT receptive fields is unrelated to the simple/complex distinction commonly used for V1 receptive fields.

### Simple receptive fields

**Figure 2:**
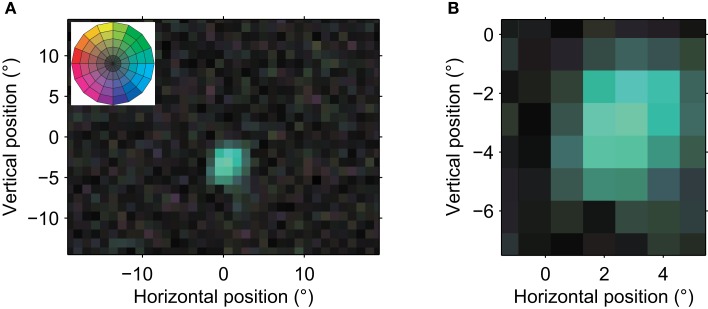
**Receptive field of an MT neuron with a single preferred direction**. **(A)** A global view of the RF. Each pixel in the image corresponds to a 1° × 1° patch on the retina. The fovea is at coordinate (0,0). The hue of each pixel corresponds to the preferred direction at that location while the color saturation indicates the strength of the direction preference (see color wheel in Figure 1F, and inset). These graphical conventions are the same for all color coded receptive field maps in this paper. This neuron’s RF had an eccentricity of ∼5°, and a diameter of ∼5°. The reverse correlation analysis used 23,890 spikes recorded in 11.5 min, and the isolation quality was high (*L*-ratio = 5.8 × 10^−5^). The low saturation outside the single contiguous area shows that only a single, restricted region of visual space consistently led to spikes from this neuron; its spatial receptive field was contiguous. **(B)** This close-up view of the receptive field shows the relatively homogenous direction preference within the receptive field. None of the preferred directions differed significantly from each other (bootstrap, *p* > 0.05). This neuron had a strong preference for motion to the right (blue-green) in the center of its receptive field. The strength of direction tuning gradually decreased toward the edges of its RF. The properties of this neuron corresponded well with the classical view of an MT neuron as a detector of a single direction of motion in a single, contiguous part of the visual field.

The MT neuron illustrated in Figure 2 preferred rightward motion and had an RF centered ∼3° below and 2° to the right of fixation (0,0). The RF diameter was ∼5°. This cell’s RF structure was consistent with the classical view of an MT RF inferred from qualitative or manual measurements (Raiguel et al., 1995; Krekelberg and Albright, 2005; Hartmann et al., 2011); it preferred the same direction of motion anywhere in its RF and the RF’s spatial profile had a single peak.

In 54% of our sample, the RF structure was similarly simple: a single preferred direction and a single spatial peak. We compared some of the basic receptive field properties to those in a previous detailed report by Raiguel et al. (1995). First, we found that RF area increased as a log-linear function of eccentricity with slope 0.032 (*R*^2^ = 0.16) and intercept 9.2°. In the Raiguel study these were 0.044 and 32°, respectively. Following (Britten and Heuer, 1999), we also examined the linear relationship between RF size (sum of the sigmas of a fitted 2D Gaussian) and eccentricity, and found a slope of 0.24 with an intercept of 1.9° (*R*^2^ = 0.18). Similar analyses in the literature report slopes between 0.43 (Tanaka et al., 1986) and 0.85 (Britten and Heuer, 1999). These 2D fits also showed that RFs were typically elongated (median ratio of the long to the short axis was 2.7) and the preferred direction was typically perpendicular to the long axis of the spatial RF (mean angle 98°, Rayleigh test, *p* < 0.001). Raiguel et al. (1995) have previously reported similar properties in area MT neurons. Quantitatively, our reverse correlation approach appears to result in slightly smaller RF estimates; however, we take the qualitative match as evidence that – when a receptive field is simple – our reverse correlation RF mapping method measures geometrical properties that are similar to those measured by more traditional methods.

### Multiple preferred directions

**Figure 3:**
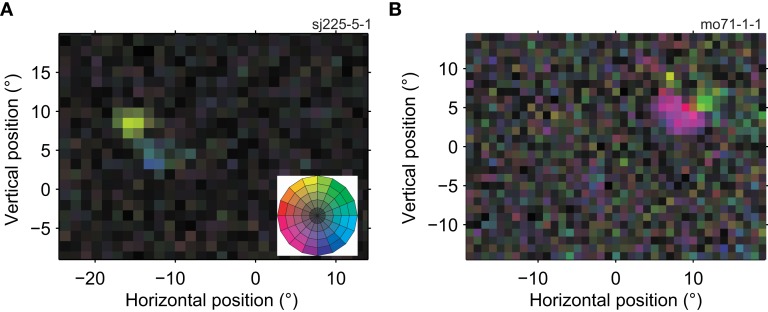
**Receptive fields of two MT cells with multiple preferred directions**. The conventions in this figure are the same as in Figure 2A, the color wheel inset serves as a reminder of the mapping between direction and color (see Figure 1F). **(A)** This neuron preferred upward (yellow) and rightward (blue) motion in two adjacent regions of its RF (11,450 spikes; 15 min; *L*-ratio = 0.001). **(B)** This neuron preferred left and downward (purple) and upward (light green) motion in two adjacent regions of its RF (6413 spikes; 14.4 min; *L*-ratio = 0.057). These RF maps show that direction preference of neurons in MT depended on the location within the receptive field.

Figure 3 shows two example neurons whose RF reveals a departure from the standard model of a unidirectional MT receptive field. The neuron in Figure 3A preferred upward motion in one region (yellow), and rightward motion in an adjacent region (blue). The neuron in Figure 3B had sub-regions with preferences for motion left and slightly down (purple) and up and slightly right (light green). Across our sample of MT neurons, we found that the RF of 38% (58 out of 151, *p* < 0.05, Bonferroni-corrected bootstrap; see Materials and Methods) had two or more sub-regions with significantly different preferred directions within their RF.

Formally this shows that many MT cells do not have a single preferred direction. However, one should not expect a real receptive field to be perfectly homogeneous, and these deviations from homogeneity could be statistically significant but functionally irrelevant. For instance, the differences in preferred direction could be small, the locations with a different preferred direction could be limited to a few pixels on the screen, or provide only minimal drive to the neuron. To assess the functional significance of the sub-regions, we divided each receptive field into two sub-regions (Using *K*-means clustering with *K* = 2; see Materials and Methods). For each RF, we calculated three quantities; the directional range (the difference in preferred direction between the sub-regions), the relative tuning strength (the ratio of the tuning strength in the weakest and strongest regions), and the relative size (the ratio of the size of the smallest and largest sub-region).

**Figure 4:**
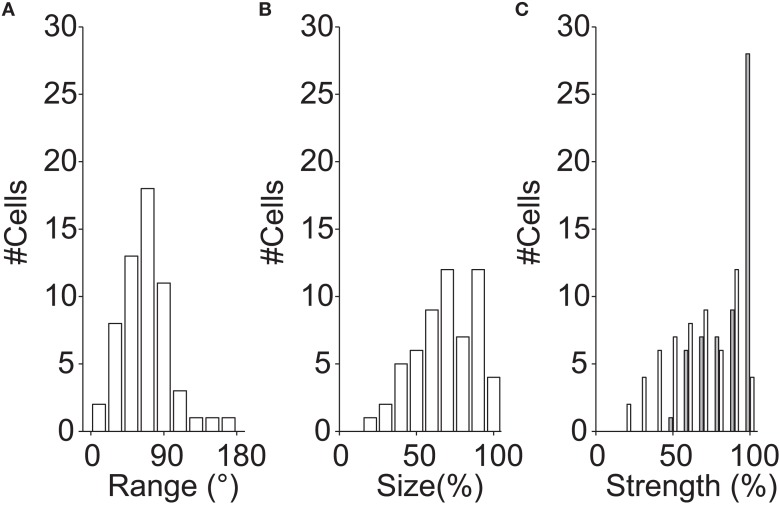
**Multiple preferred directions**. These histograms show population data for the 58 neurons with significantly different preferred directions within their RF. Each RF was first divided into two regions based on the locally preferred directions of motion (see Materials and Methods); the histograms compare properties of those two regions. **(A)** Histogram of difference in preferred direction. **(B)** Histogram of the relative size of the sub-regions. A relative size of 100% represents a neuron in which the two regions of the RF with different preferred directions had the same size. **(C)** Histogram of the relative tuning strength of the two regions (white bars). A relative strength of 100% represents a neuron in which two regions of the receptive field, with significantly different preferred directions, generated equally large responses. The gray bars represent the relative strength after normalizing by the difference in size [i.e., correcting for the effect shown in **(B)**]. Together, these data show that the differences in preferred directions were often large (but rarely larger than 90°), and that the response modulation and the size of the weaker/smaller region is typically 2/3rd of that of the stronger/larger region.

For the example cells shown in Figure 3, the directional range was 142° (Figure 3A) and 90° (Figure 3B), the relative tuning strength was 62%, and 81%, and the relative size was 100%, and 90%, respectively. Figure 4 shows histograms of these measures for the 58 neurons in our sample that had sub-regions with significantly different preferred directions (The analysis was restricted to this subset because the division into sub-regions would not be meaningful for neurons with only a single preferred direction).

Figure 4A shows that many MT neurons had sub-regions with preferred directions that differed by more than 30°, but the difference in preferred direction in the two sub-regions rarely exceeded 90°. Figure 4B shows that the size of the smaller region was typically two-third of the size of the larger region. Figure 4C shows that the tuning strength of the weakest sub-region was about two-third of the tuning strength of the strongest region (white bars). The latter effect, however, is confounded with the size of the sub-regions (larger regions are expected to have stronger tuning strengths). To disentangle these effects, we also show a histogram of the tuning strength normalized by the size of the sub-region (Figure 4C; gray bars).

**Figure 5:**
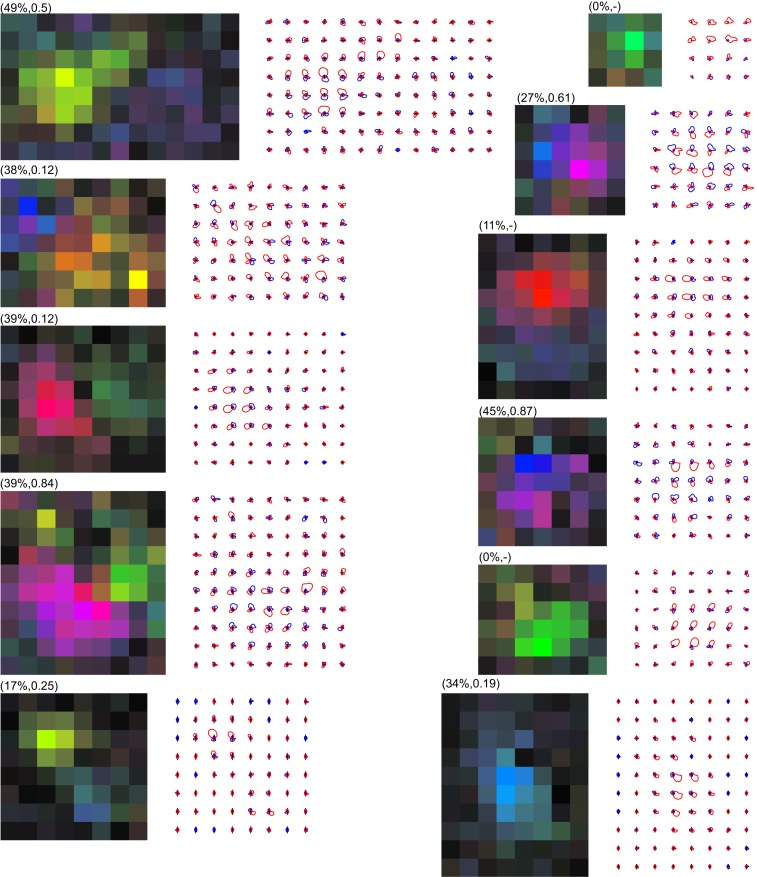
**Examples of MT neurons with multiple preferred directions**. The RF maps were calculated at a 1° × 1° spatial resolution and cropped to leave a single pixel outside the bounding box of the RF. The color maps use the representation of Figures 1E,F. The matching line drawings show separate tuning curves for excitatory (red) and suppressive (blue) effects, following the representation of Figure 1D. The labels above the color maps show the percent missing and the valley-over-peak ratio for that specific neuron (See Materials and Methods, and the “Multiple Peaks” section for an explanation of these measures). These examples show that there is a wide range of preferred direction distributions, as well as a wide range of excitatory and suppressive interactions within the RF of MT neurons. In our sample, 38% of neurons had two or more preferred directions within their excitatory RF.

Taken together these analyses support the view that sub-regions with different preferred directions could have functionally significant consequence; the differences in preferred direction were large, both sub-regions were of similar size, and both provided significant drive to the neurons. Given the complexity of these receptive fields, a straightforward and complete quantification of their properties is elusive; instead we provide additional examples (Figure 5) to provide qualitative insight into the range of RF complexities.

### Validation with a classical stimulus

The main advantage of our mapping stimulus is that it requires no assumptions about the location, size, or preferred directions within the RF; this information is all extracted after the recording. One possible concern, however is that, given the complexity of the motion pattern, the receptive field properties could be affected by interactions among multiple simultaneously present directions of motion, or interactions between center and surround stimulation.

**Figure 6:**
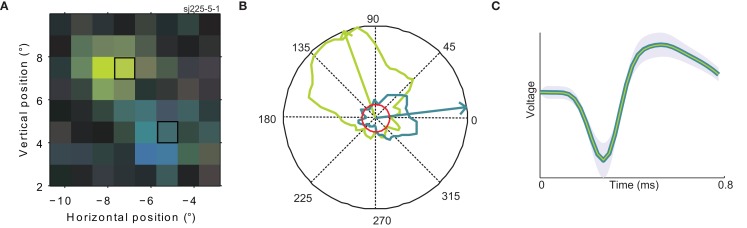
**Confirmation of multiple preferred directions**. **(A)** A close-up of the RF of the same neuron shown in Figure 3A (for a color legend, see Figure 1F). The two highlighted rectangles indicate the two regions of the RF where direction tuning was mapped with a single moving dot. **(B)** Polar direction tuning plots for single moving dots. Direction of stimulus motion is represented as the polar angle, firing rate as the radius. The maximum firing rate was 16 spikes per second. The green (blue) curve refers to the response to a single dot moving within the green (blue) highlighted patch of **(A)**. The red circle represents the response of the neuron during steady fixation of a featureless gray screen (background firing rate). Arrows represent the circular mean of the firing rate distributions; i.e., the preferred direction per patch. **(C)** Average spike waveforms evoked by stimulation of each of the patches (in colors matching the preferred direction). The green curve is deliberately thinner to make both, nearly identical waveforms visible. The shaded area represents one standard deviation. These analyses show that preferred direction changes within the RF of MT neurons are not caused by interactions due to the complex motion stimulus, but occur even for single dots moving within the RF of a single well-isolated neuron.

To address this, we validated our method and findings using a single moving dot. For a subset of cells, we selected between two and five 1 × 1° patches within the RF in which the online reverse correlation analysis suggested that the preferred directions were different. We then mapped the direction tuning curves per patch by presenting a single dot moving in one of 64 directions (PrefDir stimulus; see Materials and Methods). Figure 6A shows a close-up of the RF of Figure 3A and highlights the two patches that were chosen for the PrefDir stimulus. Figure 6B shows the direction tuning for single dots moving within each of these two patches. First, these tuning curves confirmed the description obtained from the reverse correlation stimulus; the cell preferred upward motion in the upper part of its RF, and rightward motion in the lower part. These differences were statistically significant (*p* < 0.05, bootstrap test). Second, the PrefDir paradigm confirmed that the response in both patches was excitatory; the response in either patch was higher than the baseline (red line in Figure 6B). Third, this paradigm allowed us to confirm that the different preferred directions were not caused by poor unit isolation. The green curve in Figure 6C is the average spike waveform when only the green patch was visually stimulated; the blue curve is the average spike waveform when the blue patch was stimulated. The waveforms were nearly identical and they were well separated from all other waveforms, including the noise (*L*-ratio = 0.001). This confirms that the same unit had different directional preferences in the two locations. These preferences were not the consequence of the non-linear interaction between different locations in the RF or between center and surround because they were found even when those locations were stimulated with only one dot at a time.

**Figure 7:**
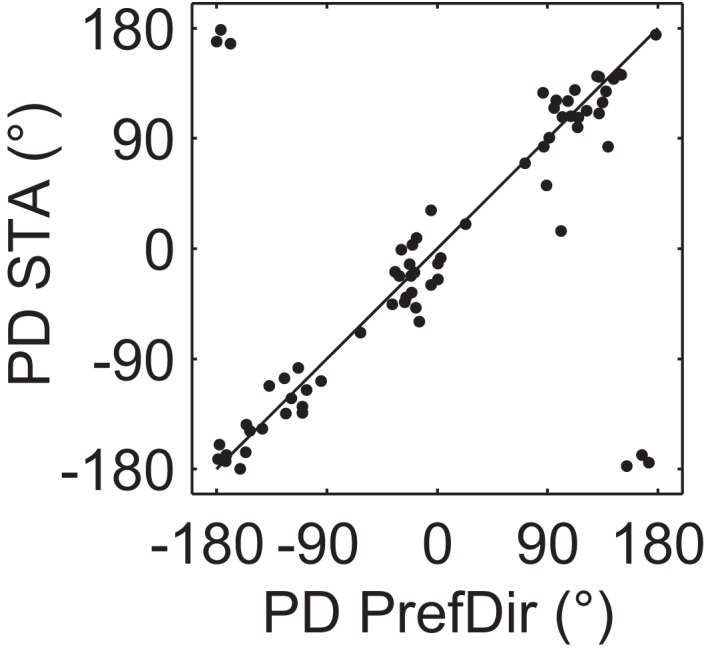
**Validation of preferred direction estimates**. Each point represents the preferred direction in one location for those neurons where the PD was mapped using a single moving dot (PrefDir; horizontal axis) as well as the full-field motion stimulus (BMDots; vertical axis). Data are from 70 patches recorded in 22 neurons. Note that the 6 “outliers” are merely the result of presenting polar data on a Cartesian plot. The circular correlation between the two estimates was 0.9 (*p* < 0.001). This figure shows that the whole-field BMDots motion stimulus and its reverse correlation analysis result in preferred direction estimates that match those obtained with a classical single dot mapping stimulus.

As a quantitative measure of correspondence between classical direction mapping (PrefDir) and our reverse correlation method (BMDots), we determined preferred direction maps for 22 neurons (18 of these had multiple preferred directions according to the reverse correlation method). Across these 22 neurons, we tested 70 patches with the single dot stimulus. As shown in Figure 7, the correlation between the PD estimate of the single dot and the PD estimate of the reverse correlation method was large and highly significant (circular correlation *r* = 0.9; *p* < 0.001). This shows that the reverse correlation analysis extracted essentially the same properties as a classical mapping stimulus. Specifically, this shows that our finding of multiple preferred directions within the RF cannot be ascribed to the unusual nature of the BMDots stimulus or its relatively complex analysis. In addition, in eight neurons we collected enough data using the single dot stimulus to confirm that the preferred directions were individually significantly different. For these eight cells, the ratio of the firing rates in response to the different preferred directions of motion in the two locations ranged from 0.43 to 0.75. Hence for these cells we can be sure that the multiple preferred directions were not artifacts of our stimulus, and that both locations provided similar drive to the neurons.

### Multiple peaks

**Figure 8:**
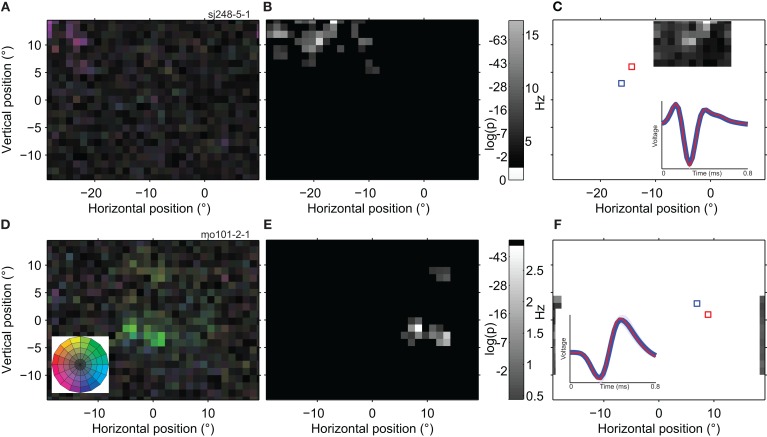
**Spatially multi-peaked receptive fields**. **(A)** Receptive field map of a single neuron with a large number of sub-regions. The analysis was based on 98,376 spikes recorded in 22 min, with a high quality isolation (*L*-ratio = 0). The receptive field was to the left and above the fovea, and spanned a total range of ∼15°. The general direction preference was down and to the left [purple; see color wheel inset in **(D)**]. **(B)** Statistical analysis of the RF map in **(A)**. The gray scale in this map reflects the significance of direction tuning (the log of the *p*-value of the Rayleigh test). Locations outside the estimated RF (see Materials and Methods) are shown in black. **(C)** Response of the same neuron as in **(A,B)** to a single dot moving in the preferred direction within a 1 × 1° patch (using the GridRF stimulus; see Materials and Methods). Here the gray scale represents the average firing rate of the neuron. Unmapped parts of the visual field are shown in white. The inset shows the average spike waveform evoked by stimulation of red and blue outlined parts of the receptive field. Shading represents one standard deviation; in some places it is narrower than the line representing the average wave form. The red curve is deliberately thinner to make both, nearly identical, waveforms visible. **(D)** The receptive field of a second example neuron (20,592 spikes; 27 min; *L*-ratio = 0.002). This neuron also has multiple sub-regions, and prefers a different direction of motion in the lower (green; up and to the right) than in the upper region (yellow; up and to the left). **(E)** Statistical analysis of the direction tuning of the neuron shown in **(D)**, same conventions as **(B)**. **(F)** Single dot response for the neuron shown in **(D,E)**, same conventions as **(C**; The upper satellite of the RF was not mapped due to the restricted size of the GridRF stimulus). These examples show that individual, well-isolated MT neurons can have widely spread, multi-peaked receptive fields and that this substructure is independent of the stimulus used to map it.

Figure 8 shows two example cells in which highly responsive spatial sub-regions (bright patches) were separated by regions with much reduced responsivity (dark patches). Figures 8A,D quantify the actual responses across the whole screen, but to highlight the sub-regions, we applied a statistical threshold for direction tuning in Figures 8B,E. The cell in the top row of panels appears to have seven isolated sub-regions or peaks, each of which prefers motion down and to the right. The bottom row of panels shows data from a different cell, which has two sub-regions close together and a third that is more than 10° away from the main RF location.

Qualitatively, these patchy RFs do not fit with the view of the RF as a single hill of sensitivity. Quantitatively, however, it is not clear which model one should test these RFs against. Testing whether they are well fit by a parametric model (e.g., 2D Gaussians), seems overly restrictive, hence the lack of fit would be non-informative. In the absence of a formal definition of the shape of an RF, we performed four somewhat interrelated analyses of the RF properties that, taken together, quantify that these RFs are not well-described by a single hill of sensitivity.

**Figure 9:**
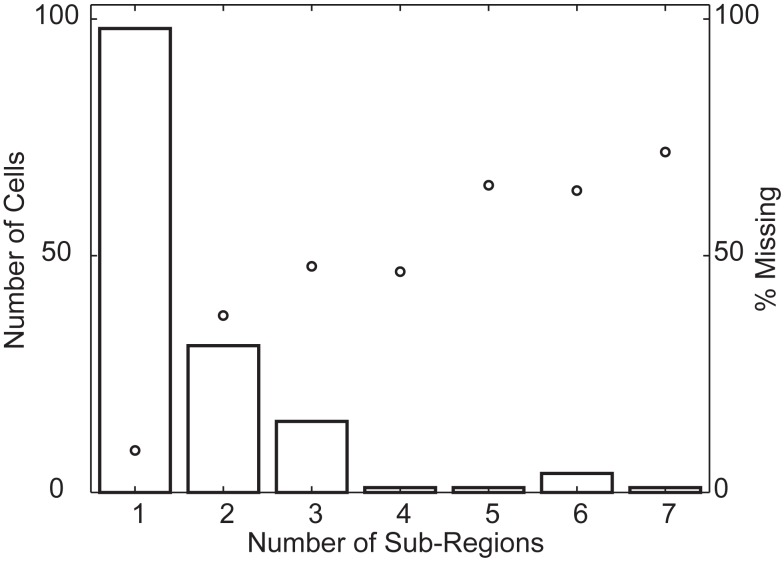
**Population overview of multi-peaked receptive fields**. The histogram (using the vertical axis on the left) shows the number of cells whose receptive field is composed of a given number of sub-regions (horizontal axis). The circles (using the vertical axis on the right), represent the fraction of space inside the convex hull of the receptive field where cells with a given number of sub-regions (horizontal axis) fail to cross the response threshold. This shows that a considerable fraction of cells have receptive fields with multiple, spatially separate sub-regions with high sensitivity.

First, we determined how sensitivity changed when moving away from the peak of the RF. We found that 36 (24%) of the RFs were significantly non-monotonic (*p* < 0.05, see Materials and Methods). We refer to cells that pass this statistical test as “multi-peaked.” Second, we counted the number of peaks in the RF by counting the segments in an RF map thresholded at a *p* < 0.01 significance level for direction tuning. The histogram in Figure 9 shows that most cells responded to visual stimuli in a single region of space (one sub-region), but a large fraction (53/151) contained two or more sub-regions of high sensitivity, separated by regions where sensitivity was below the threshold. In principle, this analysis depends on the arbitrary threshold that we used to define a significant response. For the range of practically relevant thresholds (i.e., for *p-*values smaller than 0.01), however, we found that these findings were robust. Third, we determined each RF’s convex hull and then determined the fraction of space inside the hull where the response failed to cross an arbitrary threshold (*p* < 0.01). We refer to this as the “percent missing.” Figures 5 and 11 list the percent missing for each of the examples, and the average percent missing is shown as the circles in Figure 9. Neurons with an RF consisting of a single sub-region on average responded to more than 91% of their RF’s convex hull (9% missing). On average, however, the cells with two or more sub-regions were unresponsive to between 36 and 70% of the area within their convex hull. For the subset of multi-peaked cells, the median percent missing was 27%. Qualitatively similar results were obtained for other practical, but arbitrary choices of the statistical threshold (i.e., below *p* < 0.01; not shown).

**Figure 10:**
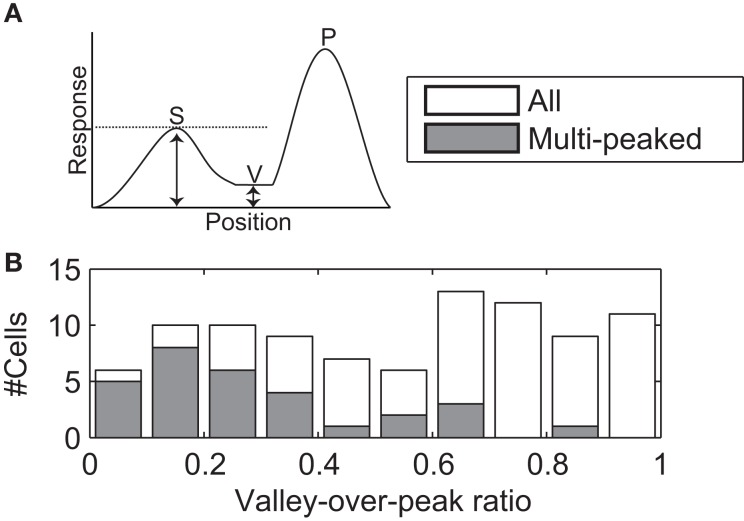
**Valley-over-peak analysis**. **(A)** Schematic of the analysis. P indicates the primary peak (highest sensitivity in the RF), S the secondary peak and V (Valley) is the lowest sensitivity on the line between P and S. The valley-over-peak ratio is defined as the ratio of the tuning strength at V and the tuning strength at S. **(B)** Histogram of the valley-over-peak ratio for 93 cells. The shaded bars represent the cells that were significantly multi-peaked (i.e., non-monotonic). This analysis shows that, for multi-peaked cells, sensitivity between two high sensitivity peaks on average drops to ∼20% of the response at the weaker of the two peaks.

**Figure 11:**
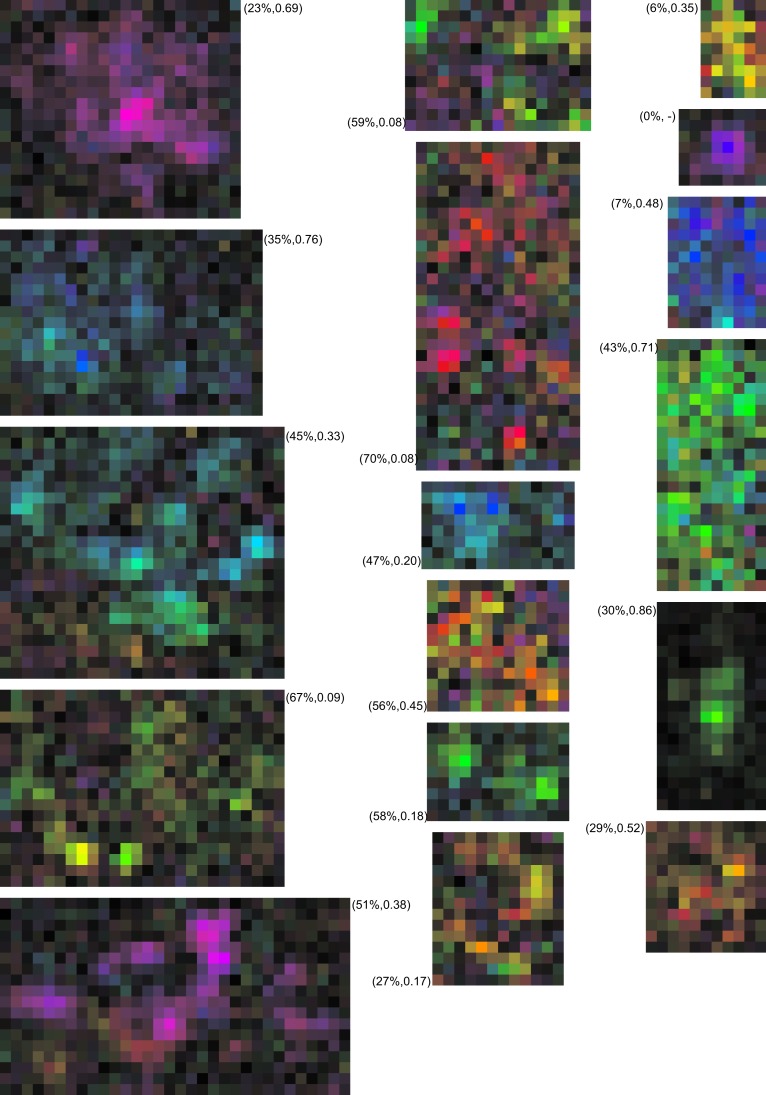
**Examples of MT neurons with multi-peaked receptive fields**. The RF maps were calculated at a 0.5° × 0.5° spatial resolution and cropped to leave a single pixel outside the bounding box of the RF. The color maps use the representation of Figures 1E,F. The labels show the percent missing and the valley-over peak-ratio for each neuron. While difficult to capture in a single, quantitative measure these examples show that a description in terms of a single hill of sensitivity does not capture the complexity of the MT RF. In our sample 24% of neurons had such non-monotonic receptive fields.

All three measures suggest that regions of low sensitivity were intermixed with regions of high sensitivity, but they do not quantify how low the sensitivity can drop. If sensitivity only dropped by a few percent, the functional consequences of these inhomogeneities could be unimportant. To quantify the drop in sensitivity, we determined the two highest peaks of sensitivity in the receptive field, and found the lowest sensitivity on a line connecting the peaks (i.e., the valley floor, see Figure 10A). To compare across neurons we expressed the tuning strength in the valley as a fraction of the tuning strength of the weakest (i.e., second) of the two peaks. We refer to this as the valley-over-peak ratio; Figures 5 and 11 list this ratio for each of the example cells, and a histogram for all cells is shown in Figure 10. For the multi-peaked neurons, the median valley-over-peak ratio was 0.21. In other words, when traveling along a line from the strongest peak in the RF to the second peak, the tuning strength first dropped to a low level from which it increased almost fivefold to the second peak.

Given the difficulty of finding a single quantitative measure that fully describes the complex spatial sensitivity pattern of these RFs, we also provide additional qualitative insight by presenting a number of example RFs in Figure 11.

#### Validation with a classical stimulus

The full-field mapping stimulus was advantageous to uncover remote parts of the RF that a local mapping stimulus might not probe. However, full-field visual stimulation raises the concern that the multiple peaks could be the consequence of non-linear interactions within the classical receptive field, or by center-surround modulation.

To assess this possibility we probed a subset of cells with a classical stimulus and analysis. We used a single dot, moving in the preferred direction at one of 121 locations in an 11 × 11 grid centered on the RF (GridRF stimulus; see Materials and Methods). Figures 8C,F represent the average firing rate following stimulation of one of the RF locations. First, the good agreement between this more traditional receptive field map, and the one obtained by reverse correlation (Figures 8B,E) confirms that the multiple peaks were not a mere consequence of our full-field reverse correlation mapping. Second, this analysis also confirms that multiple peaks arose at least partially due to low firing rates evoked from some areas, and not only a lack of direction selectivity. Third, we also used this paradigm to confirm that the separate sub-regions did not arise from the poor isolation of separate neurons. The average waveforms evoked by stimulation of individual patches (insets in Figures 8C,F) were almost identical, thus confirming that the same well-isolated neuron responded to each of the two patches.

We quantified the similarity between the spatial tuning profiles obtained with the more traditional stimulus and those obtained with the BMDots stimulus. For the cells in Figure 8, the similarity index (see Materials and Methods) was 0.93 (Figure 8A) and 0.89 (Figure 8D). The median similarity across 61 cells was 0.92. This again indicates that our reverse correlation method and the single dot response maps measure very similar properties. Of these 61 cells, 20 cells were significantly multi-peaked. For those cells we repeated the percent missing analysis now using the firing rate as a measure of tuning strength. The median percent missing based on the responses to the single dots was 20%. This was not significantly different (*p* > 0.8) from the median percent missing (27%, see above) determined with the full-field BMDots stimulus. Moreover, across the sample of neurons, the percent missing determined with single dots was significantly correlated with the measures obtained with the BMDots stimulus (*r* = 0.54, *p* < 0.05). Repeating the valley-over-peak analysis with the single dot stimulus showed that the valleys for single dots were less pronounced than those measured with the full-field stimulus (0.77 vs. 0.21, *p* < 0.05). Possible explanations for this difference are presented in the section “Discussion.”

#### Scotomas?

**Figure 12:**
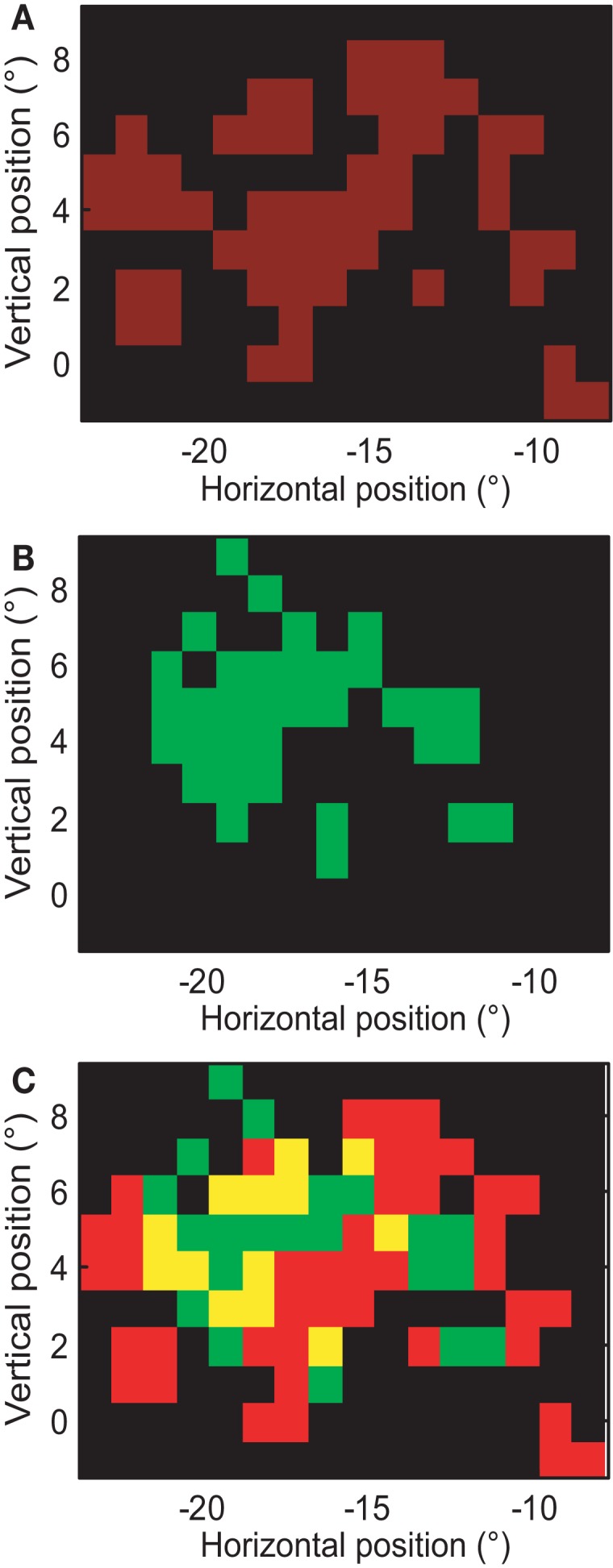
**Gaps in the receptive fields are not caused by retinal scotomas**. **(A)** Close-up of the receptive field map for the neuron whose complete receptive field is shown in Figure 8A. Red pixels represent the estimated RF and black pixels represent regions outside the RF. **(B)** Receptive field of a neuron recorded from the same hemisphere of the same animal. Here the color green is used to identify the receptive field. **(C)** Overlay of **(A,B)**. Yellow patches represent the locations where both cells responded significantly. Some patches in which the neuron of **(A)** failed to respond, did evoke a response in the neuron of **(B)**, hence the gaps in these RFs could not have been caused by retinal scotomas.

A relatively uninteresting explanation of multi-peaked RFs in MT could be the presence of scotomas in V1 or the retina. If this were the case, RFs covering the same retinal position in the same animal should have the same gaps. To test this, we overlaid the RF of two neurons with multi-peaked RFs, recorded in the same animal and same hemisphere (sj239-012-1, sj248-005-1): see Figure 12. The RF of the first cell covered some of the gaps in the RF of the second cell. This finding was typical for cells with multi-peaked receptive fields; hence, the gaps were not due to the absence of retinal input, but because the particular MT neuron failed to sample that input.

For completeness, we note that – except for their RF properties – the cells with conventional and non-conventional RFs did not form qualitatively different groups. For instance, we found no evidence of spatial clustering and the firing rate did not depend on the receptive field properties. Across cells with conventional RFs the firing rate was 16 ± 13 spikes/s (mean and standard deviation across the sample), while cells with non-conventional RFs responded with 21 ± 20 spikes/s. This difference was not statistically significant (*p* = 0.51, rank sum test). Cells with multiple preferred directions, however, were also more likely to have gaps in their receptive fields than cells that did not have multiple preferred directions (χ^2^ test for independence, *p* < 0.01).

## Discussion

We developed a technique to map receptive fields with high resolution in the spatial and direction domain and used it to gain quantitative insight into the fine structure of MT receptive fields. Approximately half of the receptive fields in area MT were homogeneous; they consisted of a single region in which a single direction of motion was preferred. The other half of the neurons, however, deviated significantly from this classical view. A large fraction (38%; 58 out of 151) of cells preferred different directions of motion in different spatial locations. The differences in preferred direction were typically between 30 and 90°, and the sub-regions with different preferred directions were of qualitatively similar size and provided qualitatively similar drive to the neuron. This suggests that these differences could be important functionally and are unlikely to be mere biological variability. A smaller percentage of our sample of MT cells (24%; 36 out of 151) had multi-peaked spatial receptive fields; regions of high sensitivity were isolated from other regions of high sensitivity by regions in which sensitivity dropped on average by a factor of 5.

These results show that receptive fields in MT are more complex than commonly thought. We will first discuss our data in the light of previous findings; address a number of possible experimental confounds, and then speculate on the functional role of these neurons.

### Receptive field in homogeneities

Previous reports have claimed that MT cells prefer a single direction of motion (Maunsell and Van Essen, 1983; Albright, 1984; Krekelberg, 2008) and this preference is maintained across the spatial extent of the RF. Our results are not inconsistent with those data but simply reveal structure that these earlier methods could not detect because they used either relatively coarse hand mapping techniques, or compared only preferred and anti-preferred directions across the RF (Raiguel et al., 1995).

Our data show that some MT RFs have hot spots of sensitivity, separated by regions of low sensitivity. Given our stimulus, analysis method, and statistical thresholds, these regions of low sensitivity appeared as gaps in the receptive field. However, we cannot prove that these were truly gaps; given the appropriate stimulus, or a longer recording, it is conceivable that the neurons could be shown to respond to those parts of the RF. In other words, what we proved statistically is that the sensitivity in the receptive field did not drop off monotonically from the peak. Receptive fields with multiple peaks or hot spots have been reported in the superior colliculus (Carrasco et al., 2005), the lateral geniculate nucleus (Tavazoie and Reid, 2000), and primary visual cortex (Jones and Palmer, 1987; DeAngelis et al., 1993). These multiple hot spots were interpreted as developmental errors, as experimental noise, or not discussed at all.

While we cannot exclude the possibility that the RF inhomogeneities are errors in the developmental fine tuning of connectivity, the growing evidence in favor of such complexity suggests otherwise. Moreover, there are potential advantages to complex RFs; they are discussed below.

### Unit isolation

Multiple preferred directions of motion or multiple peaks in a single RF would not be surprising if the spikes were in fact generated by multiple cells. To address this issue, we used isolation criteria (see Materials and Methods) that minimized the erroneous classification of multi-unit activity as single units. The fact that in all our recordings very few (=0.1%) inter spike intervals were less than 1 ms supports the claim that we isolated the waveforms of a single neuron. Furthermore, the control stimuli (GridRF and PrefDir) allowed us to inspect spike waveforms evoked by visual stimulation of separate locations within the receptive field. These waveforms were indistinguishable from each other. Finally, if poor unit isolation contributed to the finding of multiple preferred directions of motion, one would expect a correlation between the quality of unit isolation and the degree to which a unit is considered to have multiple preferred directions, or a multi-peaked receptive field. To test this, we determined the correlation between the quality of isolation [as quantified by the *L*-ratio (Schmitzer-Torbert et al., 2005)] and the *p*-values associated with the tests for multiple preferred directions, and monotonicity, respectively. We found no significant correlation in either case (multiple directions: slope = 0.02, *p* = 0.31 monotonicity: slope = 0.004, *p* = 0.84). This confirms that the multiple preferred directions and multiple peaks in the MT receptive field cannot be ascribed to poor single unit isolation.

### Center-surround and non-linear interactions

The multiple directions of motion in our full-field stimulus could interact in a non-linear fashion to drive the neuron’s response either by non-linear interactions within the RF (Recanzone et al., 1997; Britten and Heuer, 1999) or by center-surround interactions (Raiguel et al., 1995; Xiao et al., 1997; Perge et al., 2005). We performed a number of analyses to determine whether such interactions could explain our findings.

First, we restricted all analyses to regions of the visual field that were predominantly excitatory (see Materials and Methods). Hence, the multiple preferred directions and multiple peaks we found are not part of a suppressive surround. However, with the full-field mapping stimulus that always covers the whole screen it is not possible to distinguish between spatial locations where motion drives a neuron (the classical RF) and spatial locations that only modulate the firing rate (the surround). Hence, in principle, these locations could be part of an excitatory modulatory surround. Our control stimuli, however, only ever stimulated a single location on the screen. Hence, for those recordings we can be certain that the multiple preferred directions and multiple peaks are neither due to non-linear stimulus interactions, nor center-surround modulation. The high degree of similarity between the single dot and the full-field stimulus analyses suggests that the two methods map the same properties and we conclude that the multiple preferred directions and multiple peaks are not the consequence of non-linear interactions among multiple stimuli or center-surround modulation. Please note that this only means that these phenomena are distinct; as we illustrate below, there may well be a single underlying mechanism.

The classical (single dot) results and the full-field results differed only in the valley-over-peak analysis. There, the regions of low sensitivity identified by the full-field stimulus were more pronounced than those identified by the single dots. One interpretation of this difference is that the non-linear interactions present in the full-field stimulus contribute to the reduced sensitivity in some parts of the RF. We note, however, that the full-field analysis can only measure the strength of direction tuning, whereas the single dot analysis quantifies the firing rate for a dot moving in the preferred direction. Hence, an alternative interpretation is that regions of low sensitivity have strongly reduced directional tuning but only weakly reduced responses in the preferred direction. This interpretation is consistent with the general agreement between the RF shape measured with the full-field and single dot stimuli, as well as the match between the percent missing analyses for single dots and full-field stimuli.

### Mechanism

**Figure 13:**
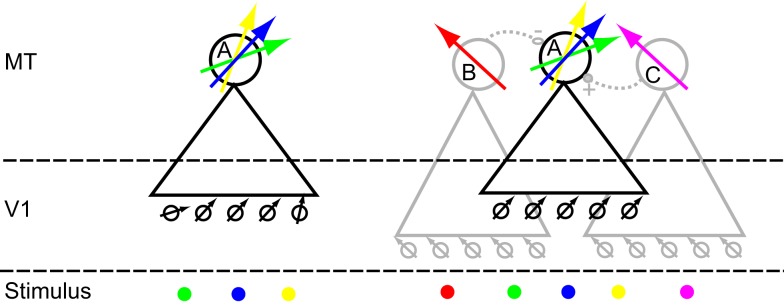
**Cartoon models of two mechanisms that could underlie the in homogeneities we observed**. Large circles represent MT neurons, labeled (A,B and C). Triangles represent their receptive field. Neuron A is the neuron whose responses we are trying to understand. The arrows represent the preferred direction for single moving dots. Small circles represent V1 neurons; black and gray arrows their preferred direction. At the “Stimulus” level, each colored dot corresponds to a particular location on the screen. The preferred direction arrow corresponding to that location is colored correspondingly. Lateral connectivity (dashed lines) between MT neurons can be excitatory (solid circle, +) or inhibitory (open circle, −). On the left is a model in which directional inhomogeneities arise through judicious sampling from the V1 population; we call this the afferent model. On the right is a model in which lateral interactions within MT create complex directional inhomogeneities; we call this the lateral model. See main text for a full description.

Circuit models that could potentially explain the complex RF structure we observed in MT are relatively easy to construct. In the simplest model (Figure 13, left; *afferent* model), the V1 neurons that provide input to the MT neuron have different preferred directions. For instance the V1 neurons that provide input on the left side of the RF (green location) have a more clockwise preferred direction than those that provide information on the central (blue) or right side of the RF (yellow). As a consequence, the preferred direction of the MT neuron depends on the location of the dots in the RF. We are not aware of any studies that quantitatively compared the direction preference of V1 neurons with the direction preference of their monosynaptic targets in MT. *A priori*, however, it does not seem far-fetched that V1 neurons with different preferred directions could project to the same MT neuron. Moreover, because our stimuli were always presented binocularly, differences in afferent preferred directions could also result from the separate inputs from the two eyes (Van Sluyters and Stewart, 1974; Zeki, 1974).

In the second cartoon model (Figure 13, right; *lateral* model), each MT neuron samples from V1 cells with the same preferred direction, but MT neurons are connected laterally to other MT neurons with different receptive field properties. The RFs of the laterally connected neurons (B and C: gray triangles) partially overlap with the RF of the neuron from which we record (A: black triangle). In this model a dot in the blue location only drives A and is not subject to lateral interactions (it is outside the RF of B and C). As a consequence, the preferred direction in the blue location reflects the preferred direction of the V1 neurons that provide the afferent input to A.

A dot in the green location drives A directly, but also indirectly through lateral interaction with B. Because B inhibits A, the response of A to motion in the preferred direction of B is reduced. As a consequence the preferred direction of A in this location (green arrow) is repelled away from the preferred direction of B. Analogously, the response of A to a dot in the yellow location is affected by C. In this example, C excites A; it therefore causes an attractive shift (relative to the preferred direction of C) in the preferred direction of A (yellow arrow).

The lateral model also allows us to clarify the relationship between the RF inhomogeneities we report here and those that have been reported previously. For instance, the response to a stimulus in the blue location would be affected by a simultaneously presented stimulus in the red location (via the inhibitory connection between B and A). This is an example of the phenomenon of surround suppression (Raiguel et al., 1995; Xiao et al., 1997; Perge et al., 2005). Similarly, two stimuli presented within the receptive field (one in the blue, the other in the yellow location) would interact through the excitatory connection between A and C. This could underlie the non-linear response to multiple stimuli presented within the MT RF (Recanzone et al., 1997; Britten and Heuer, 1999; Krekelberg, 2005). Finally, such a network can also explain why the reduced response of a V1 neuron (e.g., after adaptation) can have counterintuitive consequences for the direction tuning of a downstream MT neuron (Kohn and Movshon, 2004).

The lateral model shows that even relatively simple connectivity among MT neurons can generate highly complex response properties. Depending on the spatial layout of the stimuli, this complexity can reveal itself as center-surround modulation, non-linear interactions among multiple stimuli within the RF, or the RF inhomogeneities (even in response to single dots) that we report here. These phenomena are distinct, but they could be the consequence of the same underlying recurrent circuitry. The actual connectivity in area MT is much more complex (Malach et al., 1997) than that of Figure 13. A challenge for the future is to determine whether the relation between connectivity and response properties can be inverted. Can we determine a neuron’s (effective) connectivity based on a detailed quantitative mapping of the response to single and multiple dots, presented in the center and surround of the RF?

### Neural coding

A downstream neuron connected to a single-peaked MT receptive field always responds to a single region of the visual field; the size of that region is determined by the relationship between the downstream threshold and the overall firing rate. That size could therefore be modulated by stimulus contrast (Krekelberg et al., 2006b), or internal processes such as adaptation (Krekelberg et al., 2006a), or attention (Treue and Maunsell, 1996). Regardless of this modulation, however, the single-peaked, single direction MT neuron can be interpreted with a labeled line code for direction; it represents the evidence for a single direction of motion in a single location.

However, for a neuron downstream from a multi-peaked MT neuron, changes in the overall response rate affect the number of sub-regions, and, for some MT cells, could also change the range of directions. In other words, overall rate changes could qualitatively change the meaning of the MT spikes, which contradicts the central idea of a labeled line code (Krekelberg et al., 2006b). Viewed from a more positive angle, a multi-peaked MT neuron could provide qualitatively different information to two downstream cells with different thresholds. Whether MT or its downstream areas actually use such a computational strategy is an interesting question for future studies.

Our data suggest that rather than providing only estimates of the strength of local unidirectional motion, MT cells could signal relatively complex directional patterns. Previous reports have shown that some MT neurons are tuned for speed gradients – consistent with the idea that speed preference changes across the RF (Treue and Andersen, 1996), and that MT neurons’ disparity preference can vary across the RF (Nguyenkim and DeAngelis, 2003). It is not known whether these RF complexities co-occur in individual neurons. But if they do, MT neurons could signal the presence of highly complex three-dimensional motion patterns. Theoretical work has shown that the availability of a wide variety of flow detectors could be advantageous for optic flow analysis (Koenderink and van Doorn, 1987).

## Conflict of Interest Statement

The authors declare that the research was conducted in the absence of any commercial or financial relationships that could be construed as a potential conflict of interest.
