# Erratum: The complex structure of receptive fields in the middle temporal area

**DOI:** 10.3389/fnsys.2014.00077

**Published:** 2014-05-07

**Authors:** Frontiers Production Office

**Keywords:** receptive field, motion, middle temporal area, direction tuning, visual perception

Figure 8 was printed partially cropped, due to a typesetting error. This error does not change the scientific conclusions of the article in any way. The publisher apologizes for this error and the correct version of Figure 8 appears below.

**Figure 8:**
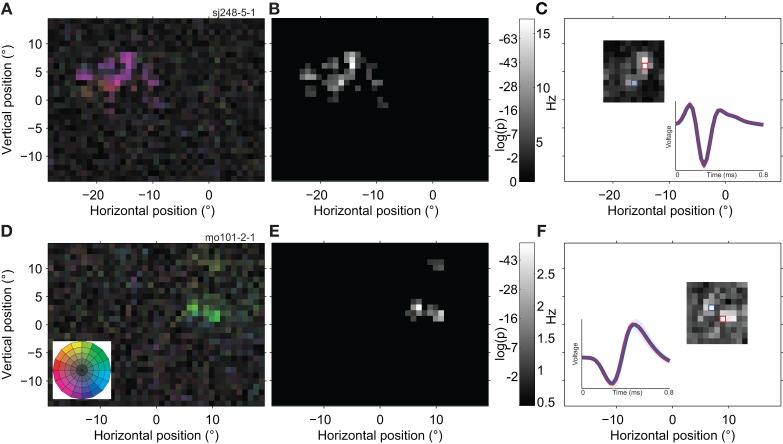
**Spatially multi-peaked receptive fields. (A)** Receptive field map of a single neuron with a large number of sub-regions. The analysis was based on 98,376 spikes recorded in 22 min, with a high quality isolation (*L*-ratio~0). The receptive field was to the left and above the fovea, and spanned a total range of ~15°. The general direction preference was down and to the left [purple; see color wheel inset in **(D)**]. **(B)** Statistical analysis of the RF map in **(A)**. The grayscale in this map reflects the significance of direction tuning (the log of the *p*-value of the Rayleigh test). Locations outside the estimated RF (see Materials and Methods) are shown in black. **(C)** Response of the same neuron as in **(A,B)** to a single dot moving in the preferred direction within a1 × 1° patch (using the Grid RF stimulus; see Materials and Methods). Here the grayscale represents the average firing rate of the neuron. Unmapped parts of the visual field are shown in white. The inset shows the average spike wave form evoked by stimulation of red and blue outlined parts of the receptive field. Shading represents one standard deviation; in some places it is narrower than the line representing the average wave form. The red curve is deliberately thinner to make both, nearly identical, wave forms visible. **(D)** The receptive field of a second example neuron (20,592 spikes; 27 min; *L*-ratio = 0.002). This neuron also has multiple sub-regions, and prefers a different direction of motion in the lower (green; up and to the right) than in the upper region (yellow; up and to the left). **(E)** Statistical analysis of the direction tuning of the neuron shown in **(D)**, same conventions as **(B)**. **(F)** Single dot response for the neuron shown in **(D,E)**, same conventions as **(C)**. The upper satellite of the RF was not mapped due to the restricted size of the Grid RF stimulus. These examples show that individual, well-isolated MT neurons can have widely spread, multi-peaked receptive fields and that this substructure is independent of the stimulus used to map it.

## Conflict of interest statement

The authors declare that the research was conducted in the absence of any commercial or financial relationships that could be construed as a potential conflict of interest.

